# Atypical Enteropathogenic *Escherichia coli* Strains form Biofilm on Abiotic Surfaces Regardless of Their Adherence Pattern on Cultured Epithelial Cells

**DOI:** 10.1155/2014/845147

**Published:** 2014-05-06

**Authors:** Hebert F. Culler, Cristiane M. Mota, Cecilia M. Abe, Waldir P. Elias, Marcelo P. Sircili, Marcia R. Franzolin

**Affiliations:** ^1^Laboratório de Bacteriologia, Instituto Butantan, Avenida Vital Brazil 1500, 05503-900 São Paulo, SP, Brazil; ^2^Laboratório de Genética, Instituto Butantan, Avenida Vital Brazil 1500, 05503-900 São Paulo, SP, Brazil; ^3^Laboratório de Biologia Celular, Instituto Butantan, Avenida Vital Brazil 1500, 05503-900 São Paulo, SP, Brazil

## Abstract

The aim of this study was to determine the capacity of biofilm formation of atypical enteropathogenic *Escherichia coli* (aEPEC) strains on abiotic and biotic surfaces. Ninety-one aEPEC strains, isolated from feces of children with diarrhea, were analyzed by the crystal violet (CV) assay on an abiotic surface after 24 h of incubation. aEPEC strains representing each HEp-2 cell type of adherence were analyzed after 24 h and 6, 12, and 18 days of incubation at 37°C on abiotic and cell surfaces by CFU/cm^2^ counting and confocal laser scanning microscopy (CLSM). Biofilm formation on abiotic surfaces occurred in 55 (60.4%) of the aEPEC strains. There was no significant difference in biofilm biomass formation on an abiotic versus prefixed cell surface. The biofilms could be visualized by CLSM at various developmental stages. aEPEC strains are able to form biofilm on an abiotic surface with no association with their adherence pattern on HEp-2 cells with the exception of the strains expressing UND (undetermined adherence). This study revealed the capacity of adhesion and biofilm formation by aEPEC strains on abiotic and biotic surfaces, possibly playing a role in pathogenesis, mainly in cases of persistent diarrhea.

## 1. Introduction

Enteropathogenic* Escherichia coli* (EPEC) is a major cause of diarrhea in children in developing countries [1, 2]. EPEC is divided in two groups: typical EPEC (tEPEC) and atypical EPEC (aEPEC), differing from each other by the presence of the EPEC adherence factor plasmid (pEAF) in tEPEC and its absence in aEPEC [3]. Therefore, aEPEC is defined as* E. coli* strains that lack the EAF plasmid (pEAF) and Shiga toxin-encoding genes (*stx*) do not express the bundle-forming pilus (BFP) and produce the characteristic histopathology lesion known as attaching and effacing (A/E) on intestinal mucosa [3–6]. The A/E lesion results from intimate bacterial adherence to the enterocytes, local microvillus effacement, and accumulation of polymerized actin of the cytoskeleton underneath adherent bacteria forming pedestal-like structures [7].

Adherence assays performed with cultured epithelial cells (HeLa or HEp-2 cell lines) show that aEPEC strains often express a defined pattern known as localized-like adherence (LAL) characterized by loose clusters of bacteria adhered to the cell surface. Other adherence patterns can also be found in aEPEC: diffuse adherence (DA), where the bacteria adhere diffusely to the cell surface; aggregative adherence (AA), where the bacteria adhere to the cell surface and to the coverslip in a stacked brick pattern; localized adherence (LA_6h_), where the bacteria adhere to the cell surface as tight clusters; and undetermined adherence (UND), when bacterial adherence cannot be classified in one of these defined patterns [6]. Nonadherent aEPEC strains are also reported less often [5, 8–10].

aEPEC strains are currently among the main agents of infectious diarrhea in several countries, including Brazil [2, 10–15], more prevalent than tEPEC ones, the main bacterial enteropathogens in children in the past [2], indicating the aEPEC as emerging and truly diarrheagenic pathogens [6].

These strains have also been implicated as agents of persistent diarrhea along with* Giardia lamblia*,* Cryptosporidium*, and enteroaggregative* E. coli* (EAEC) [2, 16, 17]. The duration of diarrhea in patients infected with aEPEC is significantly longer than of that caused by other pathogens, persisting for longer periods of time in the intestine than other diarrheagenic* E. coli *[18].

Microbial biofilms are defined as complex sessile communities formed by bacterial cells embedded in a self-produced extracellular polymeric matrix consisting of exopolysaccharides (EPS), proteins, and DNA and adhered to an inert or living surface, protecting bacteria against the deleterious effects of antimicrobial agents and increasing resistance to the host immune system [19]. Biofilms have the capacity to attach to abiotic surfaces such as glass, polystyrene, and stainless steel and even biotic surfaces such as cells and tissues [20]. Due to these characteristics, biofilms may cause serious problems in medical devices, as well as in colonization and disease [21].

In this work, we investigated the capacity of a large collection of aEPEC strains to adhere and form biofilm on abiotic and biotic surfaces. The results were also analyzed considering the patterns of adhesion on HEp-2 cells, demonstrating that aEPEC is able to form biofilm on an abiotic surface independently of this phenotype.

## 2. Materials and Methods

### 2.1. Bacterial Strains

Ninety-one aEPEC strains belonging to our laboratory collection were selected for this study. Seventy-two aEPEC strains were isolated in an epidemiological study of the etiology of acute diarrhea in children and previously identified as the genotype* eae*+/EAF-/*stx*-/*bfpA*- [14] and characterized with respect to serotype (O and H antigens) and adherence to HEp-2 cells [5]. In addition, 19 aEPEC strains of classical EPEC serogroups isolated from diarrhea were included [22] in order to increase the representatives of this specific group of strains (see Supplementary Materials available online at http://dx.doi.org/10.1155/2014/845147). The EAEC prototype strain 042 (serotype O44:H18) [23] and* E. coli* DH5*α* [24] were used as high and low biofilm formation controls, respectively. The tEPEC prototype strain E2348/69 (serotype O127:H6) [25] was used for comparison means with aEPEC strains. These strains were kept at −80°C in trypticase soy broth (TSB) supplemented with 15% (v/v) glycerol.

### 2.2. HEp-2 Cells Culture

HEp-2 cells were cultivated in Dulbecco's modified Eagle medium (DMEM - Cultilab, Brazil) supplemented with 2% fetal bovine serum in 24-well cell culture plates with glass coverslips until 100% confluence. After this period, the HEp-2 cell monolayers were washed with phosphate-buffered saline (PBS) (pH 7.2) and the attached cells were prefixed with 100% methanol for at least 20 min at 0°C or kept at this temperature until use. Preparations were rinsed six times with PBS to remove the methanol before use [26].

### 2.3. Biofilm Assay with Crystal Violet (CV)

Adhesion and production of biofilm on polystyrene and glass surfaces by aEPEC strains after 24 h of incubation was assayed following the method described by Sheikh et al. [35] with slight modifications. Overnight bacterial cultures were grown in TSB under static conditions and were inoculated into fresh DMEM supplemented with 0.4% glucose (high glucose DMEM-Cultilab, Brazil) at a 1 : 100 dilution in 24-well cell cultures plates with or without glass coverslips in static conditions and in a final volume of 1 mL. These plates were incubated at 37°C for 24 h. After the incubation period, the culture medium was aspirated, and the preparation was washed with PBS. Biofilm was fixed with 1 mL of 75% ethanol per well for 10 min, washed three times to remove the ethanol, and stained with 0.5% CV for 5 min. After PBS washings, the plates were air-dried and the CV was solubilized by the addition of 1 mL of 95% ethanol per well. After 2 min at room temperature, 150 *μ*L were transferred to a microtiter plate, and the absorbance was determined with an enzyme-linked immunosorbent assay (ELISA) plate reader (Multiskan EX-Thermo Fisher Scientific, USA) at 595 nm. All assays were performed in triplicate.

### 2.4. Biofilm Assay with Colony Forming Units Counting (CFU)

Five aEPEC strains representing each adherence pattern on HEp-2 cells (LAL, LA, AA, DA, and UND) and one nonadherent (NA) strain, each of them showing the highest levels of biofilm formation by the CV assay, were selected for the CFU assay on polystyrene and prefixed HEp-2 cell surfaces. The CFU/cm^2^ counting assay after biofilm disruption [27] was performed after 1, 6, 12, and 18 days of incubation at 37°C to quantify the viable and cultivable bacteria attached to the biofilm. Overnight bacterial cultures were grown in TSB under static conditions, and inoculated into fresh medium in a 1 : 100 dilution in 24-well cell cultures plates with or without prefixed HEp-2 cells in a final volume of 1 mL. After the incubation period at 37°C for both surfaces, with a change of culture medium every 24 h, preparations were washed with PBS and lysed with PBS-1% Triton X-100 for 20 min and then serially diluted and plated on Luria-Bertani agar (LBA) for CFU counting.

### 2.5. Confocal Scanning Laser Microscopy (CSLM)

Microscopic analysis of biofilm formation on glass and prefixed HEp-2 cells was performed according to the method described by Cleary et al. [28], with modifications. Overnight bacterial cultures were grown in TSB under static conditions and inoculated into fresh high glucose DMEM medium in a 1 : 100 dilution in 24-well cell cultures plates with glass coverslips in a final volume of 1 mL. After 24 h of incubation at 37°C, as well as after 6, 12, and 18 days of incubation, with change of culture medium every 24 h, the culture medium was aspirated and the plates were washed with PBS and fixed with 4% formalin in PBS, for 18 h at 4°C. The plates were washed again with PBS containing 0.2% of bovine serum albumin (PBS-BSA), permeabilized with PBS containing 0.1% Triton X-100 for 5 min, and washed with 0.2% PBS-BSA. Preparations were incubated with propidium iodide (PI - Molecular Probes, USA) (1 mg mL^−1^) at dilution of 1 : 1000 in 0.2% PBS-BSA, for 45 min. After four times washing for 5 min in 0.2% PBS-BSA, preparations were examined under CLSM (LSM 510 Meta, Carl Zeiss, Germany), using a 570–719 filter and wavelength of 543 nm and 1,000x magnification. For the prefixed HEp-2 cell assay, previously prepared plates were inoculated with 1 : 100 dilutions of bacteria grown overnight in TSB. Fresh high glucose DMEM medium was added in a final volume of 1 mL per well. The next steps were carried out according to the method described above, with the difference being that the preparations were incubated with fluorescein isothiocyanate (FITC)-phalloidin (Sigma-Aldrich, USA) (0.05 mg mL^−1^ in PBS) at dilution of 1 : 100 in 0.2% PBS-BSA and PI at dilution of 1 : 1000 (in 0.2% PBS-BSA) for 45 min. After four times washing during 5 min in 0.2% PBS-BSA, preparations were examined under an LSM 510 Meta (Carl Zeiss, Germany) confocal microscope using a BP500-530IR filter and wavelength of 488 nm for FITC, and a 570–719 filter and a wavelength of 543 nm for PI.

### 2.6. Statistical Analysis

Statistics were performed using GraphPad Prism 4.00 software. Differences were not considered significant when *P* > 0.05, by the Student's *t* test.

## 3. Results

### 3.1. CV Analysis of Biofilm Formation on Abiotic Surface

Indirect detection of biofilm formation was based on OD_595_ readings and analysis of mean values of assays was performed in triplicate. The mean values of OD_595_ for high (EAEC strain 042) and low biofilm production (*E. coli* DH5*α*) were 1.262 ± 0.0361 and 0.018 ± 0.0035, respectively. Strains showing OD_595_ readings exceeding the mean plus three standard deviations of the low biofilm forming control (i.e., ±0.0285) were considered biofilm formers [29].

The OD_595_ readings obtained from assays with bacteria grown either on polystyrene or glass surfaces were similar, despite the different surface properties. Since these results showed no statistically significant differences (*P* > 0.05), only the data obtained on polystyrene are presented.

**Figure 1:**
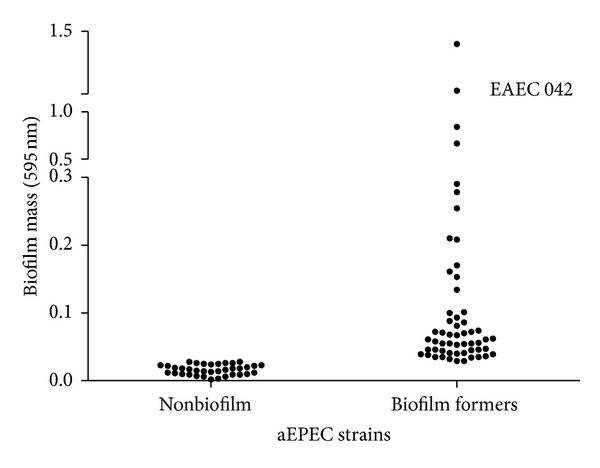
Mean absorbance at 595 nm of biofilm mass of 91 aEPEC strains on polystyrene surface after 24 h of incubation at 37°C, using the CV assay.

Of the 91 aEPEC studied, 55 strains (60.4%) were considered biofilm forming strains, whereas the other 36 (39.6%) were considered nonbiofilm forming strains (Figure 1). Of the biofilm forming strains, 27 (49.1%) were weak biofilm producers (0.0285 < OD values ≤ 0.057 at 595 nm), 16 (29.1%) were moderate producers (0.0057 < OD values ≤ 0.114), and 12 (21.8%) were strong producers (OD values > 0.114), according to the criteria of Stepanović et al. [29].

EAEC strain 042 (control for high biofilm production) formed a thicker biofilm in comparison to most of the aEPEC strains, except for strain BA4157, which showed higher OD_595_ readings (1.449 ± 0.126). Moreover, 41 (45.1%) aEPEC strains showed OD_595_ readings significantly higher (*P* < 0.05) than the values obtained for tEPEC strain E2348/69 (0.030 ± 0.0021).

**Figure 2:**
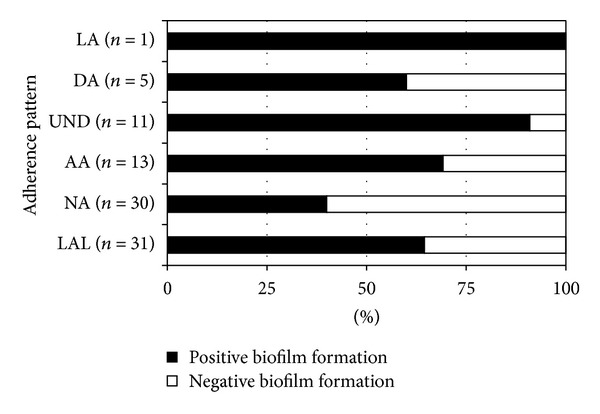
Biofilm formation by 91 aEPEC strains on an abiotic surface by CV assay and their association with adherence pattern on HEp-2 cells. LAL: localized-like adherence; NA: nonadherent; AA: aggregative adherence; UND: undetermined adherence; DA: diffuse adherence; LA: localized adherence.

These results of biofilm formation were also examined with respect to the adherence patterns of the strains used, determined in previous studies (HEp-2 cells adherence assays, supplemental data). The adherence patterns of the 91 aEPEC strains were distributed as follows: 31 LAL, 13 AA, 5 DA, 1 LA, 11 UND, and 30 NA. No association between the four defined patterns of adherence (LAL, AA, DA, and LA) and biofilm formation on an abiotic surface was observed (Figure 2). A positive association with biofilm formation was observed for the UND strains. Interestingly, 12 (40%) NA strains on HEp-2 cells were able to form biofilm. No association between the heterogeneous serogroups/serotypes and biofilm formation were observed (Table S1). Twenty-five strains (67.6%) of classical EPEC serogroups were considered biofilm forming strains, whereas 20 strains (51.3%) of nonclassical EPEC serogroups and 10 strains (66.7%) with O nontypable were considered biofilm forming.

Six aEPEC showing the strongest capacity to form biofilm and representing each adherence pattern on HEp-2 cells, as well as one nonadherent (NA) strain, were selected for further analysis: LB10 (O55:H7, LAL, OD_595_ = 0.843); BA558 (O11:H40, LA, OD_595_ = 0.070); BA4157 (ONT:H25, AA, OD_595_ = 1.449); BA2073 (ONT:H5, DA, OD_595_ = 0.071); BA2459 (O26:H11, UND, OD_595_ = 0.208); and BA2468 (ONT:H19, NA, OD_595_ = 0.290). The adherence pattern on HEp-2 cells of the others strains with the strongest biofilm formation were LAL (3 strains), AA (3 strains), and UND (2 strains).

### 3.2. CFU Counting Analysis of Biofilm Formation on Polystyrene and Prefixed HEp-2 Cells

**Figure 3:**
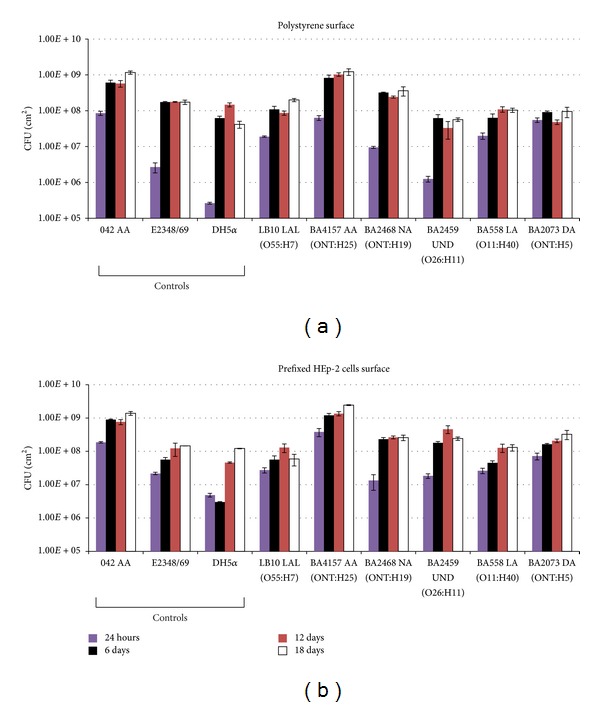
(a) Quantification of biofilm (CFU/cm^2^) formed on a 24-well polystyrene plate after 1, 6, 12, and 18 days of incubation at 37°C. (b) Quantification of biofilm (CFU/cm^2^) formed on prefixed HEp-2 cells, after 1, 6, 12, and 18 days of incubation at 37°C. The error bars represent the standard error of the mean of three replicates.

The aEPEC strains showed heterogeneous results, and no statistically significant differences in biofilm formation (*P* > 0.05) were found in relation to surface, regardless of their adherence pattern. The biomass values ranged from 10^5^ to 10^9^ CFU/cm^2^ (Figure 3). However, there was a statistically significant difference in biofilm formation (*P* < 0.05) between the CFU/cm^2^ obtained after one day of incubation in comparison with the other periods (6, 12, and 18 days) on both surfaces.

### 3.3. Confocal Scanning Laser Microscopy Analysis

**Figure 4:**
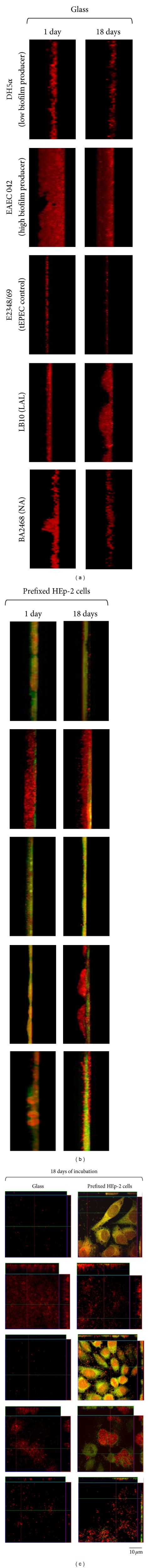
Biofilm formation by* E. coli* DH5*α*, EAEC 042, tEPEC E2348/69, and the aEPEC strains LB10 and BA2468 after 1, and 18 days of incubation at 37°C. Bacteria were stained with propidium iodide (red). Prefixed HEp-2 cells stained with phalloidin-FITC (green). Panels (a) and (b) show a* z *profile through the structure. Panel (c):* xz *and* yz *sagittal images at selected positions in the biofilm are shown at the bottom and right side of images* xy*.

**Figure 5:**
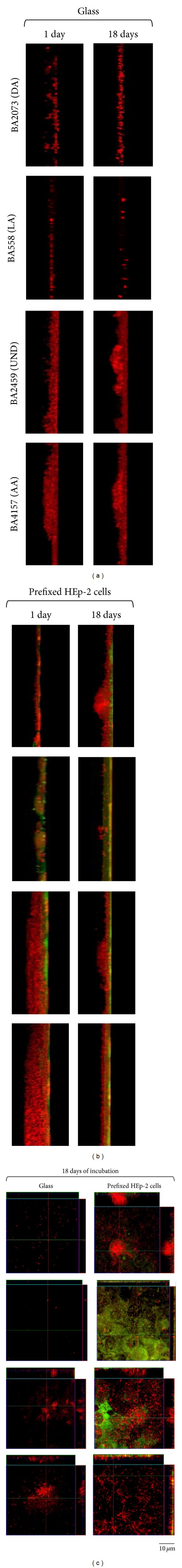
Biofilm formation by the aEPEC strains BA2073, BA558, BA2459, and BA4157 after 1, and 18 days of incubation at 37°C. Bacteria were stained with propidium iodide (red). Prefixed HEp-2 cells stained with phalloidin-FITC (green). Panels (a) and (b) show a* z *profile through the structure. Panel (c):* xz *and* yz *sagittal images at selected positions in the biofilm are shown at the bottom and right side of images* xy*.

As presented in Figures 4 and 5, some strains were able to form biofilm structures on both surfaces (glass and prefixed HEp-2 cells) (LB10, BA2468, and BA2459), while others showed higher biofilm formation on the abiotic surface (BA4157) or only on prefixed HEp-2 cell surface (BA2073).

The* E. coli* DH5*α* and tEPEC E2348/69 (LA) showed only bacteria widely dispersed on the abiotic surface, without formation of biofilm structures in all incubation periods. On the epithelial cell surface, these strains showed smaller quantities of bacteria in comparison to the abiotic surface, mainly the* E. coli* strain DH5*α*. Similarly, strain BA558 (LA) showed only dispersed bacteria for up to 18 days of incubation on both surfaces. The EAEC strain 042 (AA) formed a very compact and thick biofilm on the abiotic surface, whereas the biofilm appeared less compact and thinner on prefixed cells.

The strain LB10 (LAL) formed only a homogeneous and thin layer on the abiotic surface but with small numbers of bacteria randomly distributed on the cell surface. This strain showed a progress in biofilm formation, and, after 18 days of incubation, it was possible to observe the characteristic mature biofilm on both surfaces. Interestingly, this strain presented superior adhesion on the central region of the HEp-2 cells. The strain BA2073 (DA) showed higher preference for prefixed HEp-2 cell surface, adhering more to the central region of the HEp-2 cells, similar to the LB10 (LAL) strain. There was progressive biofilm formation only on prefixed cells, showing characteristic structure of mature biofilms, similar to pillars, at points distant from each other.

The strain BA2459 (UND) showed a compact biofilm formation formed on both surfaces for up to 12 days of incubation (data not shown). After 18 days of incubation, the characteristic structure of mature and compact biofilms, similar to pillars was visualized, mainly on the cell surface. The strain BA2468 (NA) formed clusters of bacteria for up to 12 days of incubation, and, afterwards, these clusters decreased on the prefixed HEp-2 cell surface. However, this strain showed thick biofilm formation on that abiotic surface after one day of incubation. The strain BA4157 (AA), which displayed the thickest biofilm formation detected by the CV assay, showed only a compact carpet after one day of incubation on the prefixed cell surface, similar to strain BA2459 (UND). However, after 6 days of incubation (data not shown) on the abiotic surface, it showed a compact carpet, developing pillar-like structures after a longer period of incubation.

In summary, confocal microscopy analysis of the aEPEC strains revealed highly heterogenic structures with variable depths and bacterial densities, with adhesion both on prefixed HEp-2 cells and on intercellular spaces. The strains 042, LB10, BA2073, BA2459, and BA4157 showed thicker biofilms.

## 4. Discussion

In the present work, we studied the biofilm formation ability of 91 aEPEC strains showing distinct patterns of adherence on HEp-2 cells. This pathotype is an important emerging diarrheagenic pathogen in children worldwide [10, 12, 14, 30, 31], causing acute or persistent diarrhea [32], leading to malabsorption and malnutrition [18]. Among children with persistent diarrhea, aEPEC was found to be the most common pathogen in Norway and Australia [13, 30].

Biofilm formation in the intestinal tract could contribute to colonization and disease [33]. Biofilms have been studied in* E. coli* K-12 strains [34]and in other diarrheagenic* E. coli *pathotypes [33, 35–39], whereas little is known about such phenotype in EPEC strains [27, 33]. There are few reports on the ability of aEPEC to form biofilm. Weiss-Muszkat et al. (2010) [40] found that an aEPEC strain of serotype O55:H7 has high ability to form biofilm on polystyrene surface and at the air-liquid interface at 26°C, showing potential survival strategies outside the host. The type I fimbriae is required for biofilm formation on an abiotic surface by an aEPEC strain of serotype ONT:H- [41].

Moreira et al. (2006) [27] reported that, for tEPEC strain E2348/69, adhesins such as BFP and EspA are important in microcolony formation on epithelial cells and bacterial aggregation during biofilm development on abiotic surfaces in static and in flow-through conditions. On the basis of this information, we decided to investigate biofilm formation by aEPEC strains, since, unlike tEPEC, these bacteria do not produce BFP.

The colorimetric CV assay indirectly determines the number of live and dead bacterial cells and detects the presence of polysaccharides in the extracellular matrix, based on peptidoglycan staining [42]. The ability of biofilm formation of our aEPEC strains was demonstrated on both polystyrene and glass surfaces, with no statistically significant difference between the values obtained, suggesting that biofilm formation by aEPEC strains on these two surfaces was not substratum specific. Other reports found similar results in tEPEC and EAEC strains [27, 35].

The number of biofilm forming strains (55/60.4%) was similar to the nonbiofilm forming (36/39.6%) strains after 24 h of incubation, denoting that biofilm formation by aEPEC is an intermediate virulence characteristic, allowing the successful colonization, possibly evolving to persistent diarrhea and into a state of high protection against antimicrobial drugs. On other surfaces such as glass and polystyrene, biofilms may have a role in maintenance and transmission.

aEPEC is a group of highly heterogeneous strains that may commonly exhibit the LAL pattern on cultured epithelial cells after 6 h of incubation, while some strains adhere displaying AA or DA patterns [4]. In our study, we did not find any association between the 4 distinct adherence patterns on HEp-2 cells (LAL, LA, AA, and DA) and biofilm formation on abiotic surface by the CV assay. It is worth emphasizing that the adherence pattern assay was performed with 6 h of incubation, using a specific cell lineage and employing mannose to inhibit the type 1 fimbriae. After an extended incubation period, some nonadherent strains (12/30, 40%) were able to adhere and form a thinner biofilm (NA strain BA2468), probably through interaction with type 1 fimbriae. The strains that showed an undetermined adherence pattern on HEp-2 cells (90.9%) had a high ability to form biofilm. In regard to the strains with aggregative adherence pattern, 69.2% of them formed biofilm. Indeed, the AA strain BA4157 demonstrated the highest biofilm formation on HEp-2 cells, including the EAEC strain 042, which was employed as high biofilm forming standard. The strains that did not form biofilm on abiotic surface could form biofilm on biotic surface in a substratum-specific manner, and it is possible that HEp-2 cells adherence is involved in biofilm formation on biotic surface.

CFU counting determines only the number of viable and cultivable bacteria in a biofilm [19]. The cells within the biofilm may encounter lack of oxygen and nutrients, compared to cells at the biofilm surface, and grow slowly or not grow at all. Consequently, CFU counting cannot detect them, leading to under estimation of bacterial growth [43]. The aEPEC strains formed considerably more biofilm after 6 to 18 days of incubation in comparison to the first day, denoting their adherence ability after longer periods of time. An explanation for that could be the daily changing of culture medium allowing the replacement of nutrients and discard of planktonic cells, which favors the growth of sessile bacteria. Biofilm formation of the aEPEC strains after an extended period of incubation (6 to 18 days) was evaluated to attempt to correlate it with the cases of persistent diarrhea, which can be caused by aEPEC strains [2, 30, 32].

Strain DH5*α* displayed a lower ability to form biofilm compared to the other strains only after 1 day of incubation on polystyrene surface (*P* < 0.05). The strains showed no significant statistical difference in biofilm formation (*P* > 0.05) with respect to surfaces (polystyrene and prefixed HEp-2 cell surface), producing a biomass of 10^5^ to 10^9^ CFU/cm^2^, with the exception of strains E2348/69, DH5*α*, BA2459, and BA4157, which formed more biofilm on prefixed HEp-2 cell surface after one day of incubation. The prefixed HEp-2 cells remained adhered for extended periods of incubation, when compared with the period described for living cells. According to Zepeda-Lopez and Gonzales-Lugo (1995) [26], the adherence patterns of bacteria on both living and prefixed HEp-2 cells remain the same in most* E. coli *strains.

The majority of biofilms exhibit heterogeneity in density, with cell aggregates distributed along the exopolysaccharide matrix, originating openings and channels where nutrients circulate and exchange of metabolites occurs [44]. In the gut, the passage of stool may prevent the formation of pillar-shaped structures and the biofilm may be present only on a compact carpet feature. We have seen the characteristics of a compact and mature biofilm in the strains BA2459 (UND) and BA4157 (AA) through the CLSM methodology.

Strain DH5*α*, used as a low biofilm forming pattern, confirmed this phenotype after one day of incubation. However, in flow-through continuous culture biofilm system,* E. coli *K-12 formed biofilm [34], as well as in the present study after 6 days of incubation, increasing its ability to produce biofilm after more incubation time.

In summary, no association was observed between adherence patterns on HEp-2 cells and biofilm formation in the strains studied, with the exception of those expressing the UND pattern, which showed a higher number of biofilm forming than nonforming strains. In conclusion, this study showed the ability of a large number of aEPEC strains to adhere and form biofilm on abiotic surfaces, regardless of their capacity to adhere to cultured epithelial cells. Moreover, this phenotype was independent of the expressed adherence pattern on HEp-2 cells, indicating that these strains form biofilm independently of the adhesins involved in the adherence pattern establishment. Strains displaying adherence patterns other than AA, a phenotype strongly associated with biofilm formation [35], were also able to form biofilm on both evaluated surfaces. Thus, the adherence of the aEPEC strains after an extended period of incubation may be a possible explanation for the cases of persistent diarrhea, possibly forming a biofilm on the intestinal epithelium, using the advantage of the resistance to antimicrobial agents and prolonging their survival. Their ability to form a biofilm on abiotic and inert surfaces may represent a possible transmission strategy for aEPEC to children and adults, mainly in areas with low sanitary conditions.

## Supplementary Material

Supplemental table describing the serotypes, adherence pattern on HEp-2 cells (6-h assays) and biofilm formation (OD595 nm values) of aEPEC strains and controls employed in this study. Serotypes and adherence pattern were previously determined [5, 22]. Strains were considered biofilm formers when the OD595 readings exceeded the mean plus three standard deviations of the low-biofilm forming control, i.e., ± 0.0285) [29].
