# Supplementary material for: Atypical Enteropathogenic Escherichia coli Strains form Biofilm on Abiotic Surfaces Regardless of Their Adherence Pattern on Cultured Epithelial Cells
Source: Biomed Res Int. 2014 May 6;2014:845147. doi: 10.1155/2014/845147 (PMC4032706; doi:10.1155/2014/845147)
Supplement: Supplementary file 1 — Supplemental table describing the serotypes, adherence pattern on HEp-2 cells (6-h assays) and biofilm formation (OD595 nm values) of aEPEC strains and controls employed in this study. Serotypes and adherence pattern were previously determined [5, 22]. Strains were considered biofilm formers when the OD595 readings exceeded the mean plus three standard deviations of the low-biofilm forming control, i.e., ± 0.0285) [29]. [file 845147.f1.pdf]

Table S1. Serotype, adherence pattern on HEp-2 cells (6-h assays) and biofilm formation (OD_{595nm} values) of aEPEC strains and controls

Biofilm formers strains*				Non-biofilm formers strains			
***E. coli* strain**	**Serotype**	**Adherence pattern**	**Biofilm formation (OD_{595nm})**	***E. coli* strain**	**Serotype**	**Adherence pattern**	**Biofilm formation (OD_{595nm})**
042	O44:H18	AA	1.262	*DH5α*		NA	0.017
E2348/69	**O127:H6**	LA	0.029	BA589	O5:H2	NA	0.014
BA4047	O1:H16	LAL	0.068	BA2294	O9:H33	NA	0.003
BA92	O2:H16	AA	0.093	BA179	O23:H16	NA	0.026
BA4095	O4:H45	LAL	0.039	LB4	**O26:H11**	LAL	0.026
BA558	O11:H40	LA	0.070	BA356	O33:H7	NA	0.026
BA4058	O20:H-	NA	0.035	BA 2991	O34:H-	NA	0.017
BA2103	**O26:H11**	LAL	0.072	BA 2923	O34:H6	NA	0.028
LB5	**O26:H11**	LAL	0.067	BA 1324	O34:H45	LAL	0.022
LB25	**O26:H11**	LAL	0.667	BA 442	O35:H19	NA	0.020
BA2459	**O26:H11**	UND	0.208	BA 3148	O35:H19	NA	0.014

BA 462	O51:H40	IND	0.046	BA 2964	O51:H40	UND	0.012
BA 1768	O51:H40	UND	0.044	BA 4132	O51:H48	LAL	0.012
LB10	**O55:H7**	LAL	0.843	BA487	**O55:H7**	LAL	0.023
BA320	**O55:H7**	LAL	0.032	LB8	**O55:H7**	LAL	0.006
BA1244	**O55:H7**	LAL	0.046	BA1738	O80:H26	NA	0.026
BA4147	**O55:H7**	LAL	0.068	LB2	**O86:H34**	DA	0.022
LB17	**O55:H7**	LAL	0.072	BA4013	O88:H-	NA	0.016
BA4077	O64:H23	LAL	0.081	BA2975	O88:H25	NA	0.019
BA86	O76:H19	NA	0.056	BA3443	O88:H25	NA	0.015
LB1	**O86:H34**	DA	0.053	BA2613	O101:H33	NA	0.025
BA655	O88:H25	NA	0.041	BA3160	O110:H-	NA	0.024
BA852	O88:H25	NA	0.086	LB72	**O111:H8**	LAL	0.002
BA3378	O104:H2	LAL	0.153	LB40	**O111:H9**	AA	0.023
BA2145	O105:H7	AA	0.210	BA2775	O113:H19	NA	0.007
BA4135	O108:H25	NA	0.061	LB16	**O119:H2**	LAL	0.018
BA714	**O111:H-**	LAL	0.040	LB20	**O119:H2**	AA	0.025
LB13	**O111:H9**	AA	0.062	BA580	**O119:H2**	NA	0.009

BA956	**O111:H15**	NA	0.035
BA4192	**O111:H25**	UND	0.088
BA1649	**O111:H38**	LAL	0.041
BA1887	**O111:H38**	UND	0.054
BA3690	**O111:H38**	AA	0.055
BA4009	**O114:H25**	DA	0.055
BA1444	O115:H8	UND	0.161
LB15	**O119:H2**	LAL	0.278
BA2482	**O119:H11**	AA	0.134
BA3733	**O119:H19**	LAL	0.047
BA3392	O124:H11	NA	0.088
LB2	**O125:H6**	AA	0.036
BA4182	**O125:H6**	UND	0.045
LB2	**O128:H2**	AA	0.254
LB3	**O128:H2**	UND	0.038
BA1652	O131:H4	LAL	0.061
BA3170	O145:H2	UND	0.074

BA3157	**O119:H2**	LAL	0.010
LB1	**O125:H6**	AA	0.013
LB11	**O125:H6**	AA	0.009
BA2297	O153:H11	DA	0.018
BA151	ONT:H9	NA	0.009
BA2853	ONT:H10	LAL	0.011
BA3836	ONT:H19	NA	0.028
BA3851	ONT:H38	LAL	0.010
BA3977	ONT:H45	LAL	0.006

BA585	O157:H16	AA	0.034
BA2062	O171:H48	UND	0.170
BA2073	ONT:H5	DA	0.071
BA2065	ONT:H5	NA	0.055
BA2117	ONT:H5	NA	0.058
BA1250	ONT:H6	LAL	0.101
BA2034	ONT:H10	LAL	0.046
BA2468	ONT:H19	NA	0.290
BA365	ONT:H19	NA	0.029
BA3800	ONT:H19	NA	0.029
BA4157	ONT:H25	AA	1.449
BA3574	ONT:H38	LAL	0.035

ONT, O non-typable; LAL, localized-like adherence; NA, non-adherent; AA, aggregative adherence; UND, undetermined adherence; DA, diffuse adherence; LA, localized adherence. O42, EAEC prototype strain; E2348/69, typical EPEC prototype strains; *DH5α*, K12 *E. coli*; Serotypes and adherence pattern on HEp-2 cells were previously determined [5, 22].

Strains in italic font were analyzed by CFU counting and CLSM. Serotypes in bold font belong to the classical EPEC O serogroups.

* Strains considered biofilm formers (OD_{595} readings exceeding the mean plus three standard deviations of the low-biofilm forming control, i.e., ± 0.0285) [29].
